# Editorial: Innovative 3D technologies in cancer immunity research and therapy

**DOI:** 10.3389/fimmu.2023.1235483

**Published:** 2023-06-20

**Authors:** Deepak Gupta, Boris Chichkov, Zoltan Janos Vereb, Ibrahim T. Ozbolat

**Affiliations:** ^1^ The Huck Institutes of the Life Sciences, Penn State University, University Park, PA, United States; ^2^ Engineering Science and Mechanics Department, Penn State University, University Park, PA, United States; ^3^ Cluster of Excellence PhoenixD, Leibniz University Hannover, Hannover, Germany; ^4^ Institute of Quantum Optics, Leibniz University Hannover, Hannover, Germany; ^5^ Regenerative Medicine and Cellular Pharmacology Research Laboratory, Department of Dermatology and Allergology, University of Szeged, Szeged, Hungary; ^6^ Research Institute of Translational Biomedicine, Department of Dermatology and Allergology, University of Szeged, Szeged, Hungary; ^7^ Department of Biomedical Engineering, Penn State University, University Park, PA, United States; ^8^ Materials Research Institute, Penn State University, University Park, PA, United States; ^9^ Department of Neurosurgery, Penn State University, Hershey, PA, United States; ^10^ Penn State Cancer Institute, Penn State University, Hershey, PA, United States; ^11^ Department of Medical Oncology, Cukurova University, Adana, Türkiye

**Keywords:** tumor models, cancer, bioprinting, cancer immune cell therapy, 3D technologies

Cancer still lies outside our box of understanding. The conventional methods to dissect, assemble, and replicate a human tumor artificially need to be revamped. Innovative 3D technologies offer promising potential and pave the way to develop advanced engineering methods to fight against cancer.

For decades, cancer has been a major cause of death with nearly 20 million new cases reported worldwide with a high mortality of 10 million in 2020 (1). Scientists are striving hard to understand the mechanism behind the complex interplay between cancer cells and the immune system. Still, we are far from answering the fundamental question of how cancer cells evade the immune attack (2). Current understanding states that both innate and adaptive immune cells infiltrate the tumor and influence the cancer progression through their complex network of crosstalk with heterogeneous cellular and acellular components of the tumor microenvironment. Although conventional experimental models, such as *in-vitro* 2D or *in-vivo* animal models, have laid the foundation of our understanding of cancer-immune cell interactions, such model systems fail to recapitulate the interaction of human immune system within a tumor microenvironment, and their systemic effects (3). With technological advances, the development of advanced 3D engineered models, such as organoid systems coupled with 3D bioprinting, and interconnected body-on-a-chip microfluidic devices, have shown an impressive potential to mimic natural 3D tumor-immune-microenvironment to a great extent; thus, overcoming the limitations of the conventional methods.

The Research Topic *Innovative 3D Technologies in Cancer Immunity Research and Therapy* at *Frontiers in Immunology* is dedicated to this emerging field of research. Visalakshan et al. reviewed the state-of-the-art 3D modeling approaches such as engineered organoids, 3D bioprinted systems, organ-on-a-chip, and microfluidic models for T cell infiltration, cancer resistance, and immune suppression to advance our understanding of tumor immunology thus moving a step closer towards comprehending the intricate inter-connections of the tumor-immune-microenvironment. Miebach et al.‘s review in this Research Topic targets the scope and limitations of the chicken embryo model in cancer immunological research as an alternative to time-consuming and costly mammalian tumor models. The *in-ovo* models are naturally immunodeficient, vascularized with capillaries and post-capillary venules, and possess chorioallantoic membrane accessible for experimentation. It offers a more functional natural 3D model to comprehensively study the series of events in a metastatic cascade (4, 5), tumor-immune interaction, and establish a better prognosis of cancer while circumventing the tedious process of administrative ethical approvals.

Tumor stromal cells and cancer-associated fibroblasts (CAFs) play a crucial role in tumor progression, metastasis, and therapy resistance. Still, their interaction mechanism with tumor cells remains an uncompleted puzzle. Thus, co-culturing of these heterogeneous cells and organoids offers great potential to reveal the underlying mechanisms of tumor and immune cells interactions. Yuan et al.‘s review in this Research Topic discusses recent advances, applications, and prospects of co-culture model systems designed for cancer organoids coupled with immune cells, CAR-T cells, and CAFs. There are two preeminent research articles in this Research Topic demonstrating the development of 3D engineered co-culture systems to recapitulate the tumor-immune-microenvironment. Li et al. demonstrated a 3D bioprinted model that deposits cholangiocarcinoma (CCA) surrounded by stromal cells and observed their interaction for CCA proliferation, metastasis, and drug resistance. Strating et al. developed a co-culture system for colon cancer spheroids and CAFs to study their interplay and re-organization and analyzed their effects on ECM properties and T cell proliferation. Further, to obtain an integrative picture to better understand these heterogeneous cellular interactions visually, this Research Topic contains a method article, where Elaldi et al. reported the development of a 39-antibody panel integrated with Imaging mass cytometry technology with 13 new markers to target tumor cells, immune cells, T and B lymphocytes, CAFs, ECM protein, blood and lymphatic vessels and nerve fibers.

3D Engineered systems thus provide a promising scenario in the field of cancer immunology with researchers developing new multi-functional devices to understand tumor-immune-microenvironment and to mitigate the dependence on *in-vivo* models. Nevertheless, there are several challenges yet to be addressed. The current *in-vitro* models cannot replicate the high density of natural tumors, thus making it challenging to clinically translate the therapeutic agent, engineered in a lab, to penetrate a natural tumor effectively. Further, there is ample scope to engineer the multitude of functionalities in current 3D systems to reflect the complex interactions of immune cells with other heterogeneous cellular and acellular components in tumor-immune microenvironment (6). Despite these challenges, 3D technologies offer a plethora of potential and a reliable platform for cancer immunotherapy research to accelerate drug discovery, create personalized therapies and predict clinical outcomes while gradually replacing animal testing and avoiding ethical concerns.

## Author contributions

DG drafted the editorial, and all co-editors contributed to the writing and revision of the article. All authors contributed to the article and approved the submitted version.
